# Regulated Proteolysis in *Vibrio cholerae* Allowing Rapid Adaptation to Stress Conditions

**DOI:** 10.3389/fcimb.2019.00214

**Published:** 2019-06-21

**Authors:** Nina Pennetzdorfer, Mareike Lembke, Katharina Pressler, Jyl S. Matson, Joachim Reidl, Stefan Schild

**Affiliations:** ^1^Institute of Molecular Microbiology, University of Graz, Graz, Austria; ^2^Department of Medical Microbiology and Immunology, University of Toledo College of Medicine and Life Sciences, Toledo, OH, United States; ^3^BioTechMed Graz, Graz, Austria

**Keywords:** post-translational regulation, stressor, Lon, Clp, DegS, DegP YaeL, tail-specific protease

## Abstract

The lifecycle of the causative agent of the severe secretory diarrheal disease cholera, *Vibrio cholerae*, is characterized by the transition between two dissimilar habitats, i.e., as a natural inhabitant of aquatic ecosystems and as a pathogen in the human gastrointestinal tract. *Vibrio cholerae* faces diverse stressors along its lifecycle, which require effective adaptation mechanisms to facilitate the survival fitness. Not surprisingly, the pathogen's transcriptome undergoes global changes during the different stages of the lifecycle. Moreover, recent evidence indicates that several of the transcription factors (i.e., ToxR, TcpP, and ToxT) and alternative sigma factors (i.e., FliA, RpoS, and RpoE) involved in transcriptional regulations along the lifecycle are controlled by regulated proteolysis. This post-translational control ensures a fast strategy by the pathogen to control cellular checkpoints and thereby rapidly respond to changing conditions. In this review, we discuss selected targets for regulated proteolysis activated by various stressors, which represent a key feature for fast adaptation of *V. cholerae*.

## A Brief Survey of Regulatory Events Along *Vibrio cholerae*'s Lifecycle

*Vibrio cholerae* spends much of its lifecycle outside of the host in estuarine and costal aquatic reservoirs with a geographical range from tropics to temperate waters world-wide. Along its interepidemic persistence in the aquatic reservoirs, *V. cholerae* faces temperature shifts, osmotic stress, bacterivorous predators and nutrient limitation (Lutz et al., 2013; List et al., 2018). *Vibrio cholerae* employs several strategies to cope with these numerous stressors. In particular, biofilm formation has been highlighted as a key factor for environmental survival and transmission of *V. cholerae* (comprehensively reviewed in Yildiz and Visick, 2009; Teschler et al., 2015). A central player of biofilm regulation is the transcriptional repressor HapR, which acts negatively on biofilm formation via repression of exopolysaccharide synthesis. Additionally, HapR is a quorum sensing key regulator affecting virulence factor expression and natural competence (Ng and Bassler, 2009). Since transcription of *hapR* is also activated by the alternative sigma factor RpoS, the pathways mentioned above are regulated by central physiological signals, like cell density, or carbon concentration. Biofilm-associated bacteria are generally better protected against host-derived stressors ranging from digestive enzymes, acidic pH to antimicrobial substances and exhibit hyperinfectivity in the murine model (Tamayo et al., 2010; Seper et al., 2011). Thus, biofilm clumps are a likely form by which clinically relevant *V. cholerae* initiate outbreaks (Colwell et al., 2003; Hall-Stoodley and Stoodley, 2005).

Upon oral ingestion, *V. cholerae* passages through the stomach to finally reach the small intestine, representing the primary site of colonization. Intestinal stimuli induce expression of virulence factors such as the toxin coregulated pilus (TCP) and the cholera toxin (CTX) (Childers and Klose, 2007; Matson et al., 2007). TCP represents the main colonization factor responsible for adherence to epithelial cells, while CTX constitutively activates adenylate cyclase of the host, causing a massive water efflux into the intestinal lumen known as rice-water stool (Sharp et al., 1971; Burns et al., 1983; Miller et al., 1987; Taylor et al., 1987; Herrington et al., 1988; Fishmann, 1990).

The expression of virulence factors is controlled by a complex regulatory cascade. It includes the membrane-bound transcription complexes ToxR/S and TcpP/H as well as the cytosolic transcription factor ToxT (Childers and Klose, 2007). Most of the virulence factors, e.g., CTX and TCP, are regulated by the ToxT-dependent pathway in response to intestinal stimuli, such as temperature and sodium bicarbonate (Thomson and Withey, 2014; Weber et al., 2014). In addition, ToxR can directly regulate several genes independently of ToxT. For example, ToxR inversely regulates the expression of the porins OmpU and OmpT, which plays an essential role to achieve bile resistance and full colonization fitness *in vivo* (Provenzano and Klose, 2000).

Once adapted to the intestinal conditions, *V. cholerae* starts to massively proliferate and the patient develops a severe secretory diarrhea, releasing the bacteria back into the aquatic environment. Transcriptional control of a defined set of genes at the late stage of infection facilitates the transition of *V. cholerae* into the aquatic reservoir (Schild et al., 2007). Under these conditions HapR and RpoS coordinate a drastic shift in the gene expression profile also known as “mucosal escape response” (Nielsen et al., 2006).

**Figure 1:**
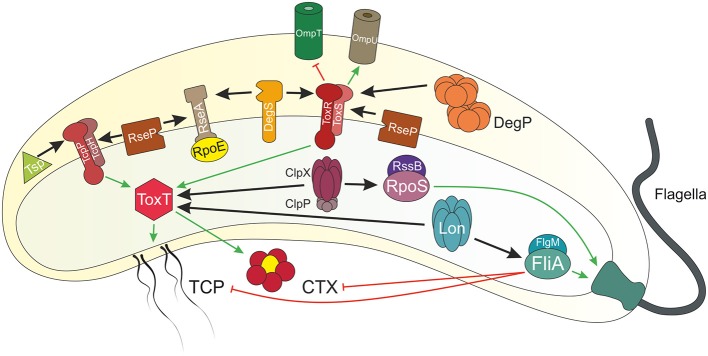
Overview of regulated proteolysis in *V. cholerae*. Shown is a *V. cholerae* cell with proteins illustrated by icons. Regulated proteolysis is indicated by black arrows. Transcriptional activation is highlighted in green and repression in red. At early stages of infection, the single polar flagellum of *V. cholerae* breaks by entering the viscous mucosal layer of the small intestine. Thereby, levels of the anti-sigma factor FlgM decreases within the cell and the alternative sigma factor FliA activates transcription of flagella biosynthesis genes. Besides of this repair mechanism, FliA also inhibits virulence genes expression, e.g., *tcp* and *ctx*. The AAA+ protease Lon degrades FliA in absence of FlgM to achieve full virulence at early stage infection. The membrane embedded transcriptional regulators and their respective partner proteins ToxRS and TcpPH activate *toxT* expression, which in turn encodes for the master regulator for transcription of the downstream genes *tcp* and *ctx*. Furthermore, ToxRS also coordinate the inverse regulation of the outer membrane porins OmpU and OmpT, in order to build up a resistance to bile salts. Both regulators are targets of regulated intramembrane proteolysis (RIP). ToxR periplasmic domain is cut by the site-1 proteases DegS and DegP, followed by site-2 protease RseP. Commensurately, Tsp mediates TcpP degradation as a site-1 protease, which in turn triggers RseP. The half-life of the virulence master regulator ToxT is controlled by the AAA+ proteases Lon and ClpXP. Additionally, the proteases DegS and RseP also act on the transmembrane anti-sigma factor RseA under envelope stress conditions, e.g., cellular or environmental changes, in order to release the alternative sigma factor RpoE to the cytosol, eventually to activate transcription of *degP, rpoE* itself or genes encoding for the T2SS. The alternative sigma factor RpoS is responsible to cope with starvation conditions, e.g., high (p)pGpp levels. At low levels of (p)pGpp, the anti-sigma factor RssB is bound to RpoS, leading to proteolysis mediated by ClpXP. At late stages of infection, RpoS is required to activate mucosal escape response by inducing the expression of chemotaxis and motility genes.

As highlighted above, adaptation to diverse conditions along the lifecycle is achieved by spatio-temporal induction of gene expression. However, termination of a regulatory pathway could be equally important to facilitate transition to the next stage of the lifecycle. An effective way to remove factors involved in gene transcription is regulated proteolysis, comprising directed degradation of defined effectors by specific proteases (Mahmoud and Chien, 2018). Indeed, regulated proteolysis has been reported as a control mechanism for several transcriptional effectors (i.e., FliA, ToxR, TcpP, ToxT, RpoS, and RpoE) along the lifecycle of *V. cholerae* (Figure 1 and Table 1), which will be discussed with an emphasis on the physiological impact and players involved.

**Table 1:** Examples for regulated proteolysis in *V. cholerae* (for details see text).

**Target for proteolysis**	**Binding partners/(anti-)sigma factors**	**Protease(s)**	**Physiological role/regulated pathways**	**Trigger for proteolysis**
FliA (σ^28^)	FlgM	Lon	Motility, virulence	Broken flagellum
ToxR	ToxS	DegS, DegP, RseP	Persistence, virulence	Alkaline pH in combination with starvation
TcpP	TcpH	Tsp, RseP	Virulence	Non-virulence-inducing conditions
ToxT	–	ClpXP, Lon	Virulence	High temperature, alkaline pH
RseA	RpoE (σ^E^ or σ^24^)	DegS, RseP	Envelope stress response	Misfolded periplasmic protein
RpoS (σ^S^ or σ^38^)	RssB	ClpXP	Motility, chemotaxis, biofilm	Non-starvation condition

## The Alternative Sigma Factor FliA (σ^28^) Is Degraded by Lon

In addition to virulence factor expression, flagella-dependent motility contributes to virulence of *V. cholerae*. The single polar flagellum is required to approach and penetrate the mucosal layer of the intestinal epithelium (Freter and Jones, 1976; Freter and O'Brien, 1981; Lee et al., 2001; Butler and Camilli, 2005). Entrance into the viscous mucosal layer puts substantial shear force on the rotating flagellum, which eventually breaks. As a result, the anti-sigma factor FlgM, usually bound to the alternative sigma factor FliA and preventing its association with the RNA polymerase, is released through the broken flagellar apparatus (Correa et al., 2004; Liu et al., 2008). Decreasing levels of FlgM result in derepression of FliA important for activation of flagella biosynthesis. Although this is an efficient feedback mechanism to sense damage of the flagellum and initiate its repair, it is dispensable in the *in vivo* setting once *V. cholerae* has penetrated through the mucosal layer. FliA inhibits virulence factor expression in *V. cholerae* by a so far unknown mechanism (Syed et al., 2009). Therefore, efficient removal of FliA from the cytosol is essential to allow full virulence expression in early stages of infection. A recent study demonstrated that FliA of *V. cholerae* is target for rapid proteolysis via the AAA+ (ATPase associated with a variety of cellular activities) protease Lon in the absence of its anti-sigma factor FlgM (Pressler et al., 2016). Under virulence-inducing conditions [i.e., *in vitro* cultivation using AKI conditions (Iwanaga and Yamamoto, 1985; Iwanaga et al., 1986)], the Lon-dependent proteolysis facilitates cholera toxin production in the presence of a damaged flagellum. Thus, the rapid removal of FliA via Lon provides a first molecular explanation for high virulence expression upon mucosal penetration at early stages of infection. Concordantly, FliA of *Escherichia coli* is also a target for Lon-mediated proteolysis and can be protected by the anti-sigma factor FlgM (Barembruch and Hengge, 2007). Thus, the FliA-FlgM-Lon feedback circuit could represent a conserved mechanism for correct flagella assembly and repair upon flagellar damage.

## Regulated Proteolysis of ToxR Is Mediated by DegS, DegP and RseP

*Vibrio cholerae* persistence and virulence are coordinated by a complex network that has been historically referred as the “ToxR-regulon” (Matson et al., 2007). ToxR is a single component signal transduction regulator comprising the N-terminal winged helix-turn-helix domain promoting DNA-binding, a transmembrane domain and the C-terminal periplasmic sensor domain (Miller et al., 1987). ToxR binds multiple AT-rich promoter proximal regions termed ToxR-boxes and is involved in transcriptional control of more than 100 genes (Miller et al., 1987; Bina et al., 2003; Goss et al., 2013). Molecular activation mechanisms for the membrane bound transcription factor ToxR are limited. Although, it has been recently shown that the transcriptional activation by the ToxR-like protein CadC in *E. coli* and binding to its operator sites, follows a model termed diffusion and capture mechanism (Brameyer et al., 2019). ToxR and TcpP (discussed below), together with their respective co-transcribed interaction partners ToxS and TcpH, are both required for maximal *toxT* expression, whereas ToxRS mediates outer membrane porin (OMP) expression directly (Higgins and DiRita, 1994; Häse and Mekalanos, 1998; Bina et al., 2003; Childers and Klose, 2007). The two major porins of *V. cholerae*, OmpT and OmpU are inversely regulated by ToxR. The *ompT* expression is repressed, whereby *ompU* is strongly induced by ToxR under nutrient rich conditions or bile salts (i.e., sodium deoxycholate), facilitating resistance toward antimicrobial compounds (Miller and Mekalanos, 1988; Provenzano et al., 2000; Mathur et al., 2007; Lembke et al., 2018). Unlike the remainder of the regulon, *ompT* is the only verified gene negatively regulated by ToxR and is derepressed under nutrient limiting conditions (Li et al., 2000). In general, *toxR* is constitutively expressed, environmental and stress stimuli may modulate the expression of ToxR regulated genes (Miller and Mekalanos, 1988). Molecular activation mechanisms for ToxR transcriptional activity are largely unknown. The two periplasmic cysteine residues (Cys236 and Cys293) of ToxR influence ToxR regulated gene expression. More specifically, DsbAB-mediated intramolecular disulfide bond and homodimer formation increase ToxR transcription factor activity (Ottemann and Mekalanos, 1996; Fengler et al., 2012; Lembke et al., 2018). Interventions in these cysteine residues (e.g., cysteine to serine substitution, reducing conditions) decrease ToxR transcription factor activity and consequently abolish the ability of proper porin gene regulation, but does not affect *toxT* transcription (Fengler et al., 2012; Lembke et al., 2018). The cysteine-reduced ToxR is a trigger to stimulate site-1 mediated proteolysis by DegS and DegP, hence resulting in ToxR degradation, most effective in strains lacking *toxS* (Lembke et al., 2018). Regulated intramembrane proteolysis (RIP) control of ToxR seems to play a physiologically important role for *V. cholerae* to properly adapt to changing environmental conditions (Almagro-Moreno et al., 2015a,b). Upon transition into a dormant stage in presence of unfavorable stress conditions, e.g., alkaline pH and nutrient limitation, ToxR becomes a substrate for RIP by the site-2 protease RseP (YaeL), which belongs to the RpoE response system (see below). ToxS also plays a major role in protecting ToxR from proteolysis under these conditions at late stationary phase (Almagro-Moreno et al., 2015b). A point mutation in ToxS (ToxS^L33S^) even triggers ToxR proteolysis comprising several site-1 proteases, including DegS, DegP, VesC, and TapA (Almagro-Moreno et al., 2015b). The two major routes of ToxR proteolysis, one responding toward the redox state and the other being sensitive to an alkaline pH and starvation, can be inhibited by bile salts, which are present in the human gut and are also known to strengthen ToxRS interaction (Midgett et al., 2017; Lembke et al., 2018). The RIP of ToxR is a highly versatile 2-step process, leading to a clearance of ToxR molecules and eventually to a termination of ToxR dependent gene regulation.

## TcpP Is a Substrate of Tsp and RseP Proteases

A second membrane-bound transcription factor that coordinates expression of *toxT* is TcpP. Like ToxR, TcpP is a bitopic protein containing a carboxy-terminal periplasmic domain and an amino-terminal cytoplasmic DNA-binding domain similar to transcription activators of the OmpR/PhoB-family (Martínez-Hackert and Stock, 1997). TcpP functions together with TcpH, a membrane protein that interacts with the periplasmic domain of TcpP. In order to activate transcription of *toxT*, ToxR recruits TcpP to the *toxT* promoter region through protein-protein interaction, where TcpP binds two pentameric repeats located between positions-53 and -38 relative to the *toxT* transcription start site (Krukonis and DiRita, 2003; Goss et al., 2010). TcpP levels in the bacterial cell are regulated both transcriptionally and post-transcriptionally. Upon entering the human intestine, environmental signals activate expression of *tcpPH* through AphA and AphB (Kovacikova and Skorupski, 1999; Skorupski and Taylor, 1999). Alternatively, under conditions that do not activate virulence gene expression, TcpP is degraded by RIP. The site-1 protease that first acts to cleave TcpP within its periplasmic domain is Tsp (tail-specific protease) (Teoh et al., 2015). Tsp is a serine protease that generally controls protein quality and gene regulation, and is rarely associated with RIP mechanisms. After the initial cleavage, TcpP becomes a substrate for the site-2 protease RseP, a membrane-localized metalloprotease that cuts within the transmembrane domain (Matson and DiRita, 2005). This cleavage and removal from the inner membrane inactivates TcpP, halting expression of *toxT* and downstream virulence genes. TcpP is normally protected from degradation through its interaction with TcpH under virulence-gene inducing conditions (Beck et al., 2004). Disruption of a periplasmic disulfide bond in TcpP results in instability of the protein, even in the presence of TcpH (Morgan et al., 2016). In addition, disruption of these periplasmic cysteines causes TcpH to also become unstable, suggesting a role for these intramolecular disulfide bonds in the TcpP-TcpH interaction (Morgan et al., 2016). Furthermore, transcriptionally active TcpP-homodimers are formed by an intermolecular disulfide bond via Cys207 in presence of the bile salt taurocholate (Yang et al., 2013). Heterodimers between TcpP and ToxR depend on the periplasmic thiol-disulfide-oxidoreductase DsbA and are enhanced by anaerobic growth conditions resulting in virulence gene induction (Fan et al., 2014). It should be noted that the outcome of ToxR and TcpP RIP is unusual in that it functions to inactivate a membrane-bound regulator and halt transcription. In the case of the RpoE-pathway (see below) and others, RIP results in transcriptional activation of downstream genes.

## ClpXP and Lon Mediate ToxT Proteolysis

ToxT was identified as a central transcription factor, activating expression of important virulence genes, e.g., encoding for TCP and CTX (DiRita and Mekalanos, 1991). ToxT is a crucial checkpoint, thereby its own synthesis is under complex control, i.e., by endogenous and exogenous factors as reviewed elsewhere (Weber and Klose, 2011). Exogenous signals negatively control ToxT activity, such as bile-derived unsaturated fatty acids (Plecha and Withey, 2015), or positively, like sodium carbonate (Thomson and Withey, 2014). Additionally, a 5′ mRNA thermometer control element of *toxT* allows access of ribosomes to the Shine-Dalgarno sequence at 37°C, but not at 20°C (Weber et al., 2014). ToxT becomes a substrate for proteolysis during virulence gene expression, reducing ToxT protein half-life (Abuaita and Withey, 2011). Precise timing of ToxT activity is crucial for the colonization success, e.g., by determining the duration and intensity of virulence gene expression. This was best monitored under *in vivo* conditions (Lee et al., 1999) and by a microarray series performed on cells grown under virulence activating conditions *in vitro* analyzing 13 time points within a 6 h period (Kanjilal et al., 2010). ToxT is part of a positive forward feedback loop and therefore positively autoregulated (Yu and DiRita, 1999). ToxT proteolysis is one mechanism to terminate its activity, which is mediated by AAA+ proteases including Lon, ClpXP, and others (Abuaita and Withey, 2011). Proteolytic instability of ToxT is regulated via stressors like high temperature and alkaline pH, and depends on an unstructured region located at amino acid positions 100-109 (Abuaita and Withey, 2011; Thomson et al., 2015). Thereby, ToxT itself harbors a protease sensitive response domain, which may trigger proteolysis depending on the listed exogenous conditions.

## The RpoE (σ^E^ or σ^24^)-dependent Stress Response Requires Proteolysis by DegS and RseP

The periplasmic protease/chaperone DegP and the membrane embedded proteases DegS and RseP are essential to react to environmental and cellular changes in Gram-negative bacteria (reviewed by Alba and Gross, 2004; Rowley et al., 2006). They are part of the envelope stress response mediated by the alternative sigma factor RpoE, first described in *E. coli* upon high temperature conditions, exposure to ethanol, or the overproduction of OMPs (Erickson and Gross, 1989; Wang and Kaguni, 1989; Mecsas et al., 1993). In absence of such stimuli, the N-terminal cytoplasmic portion of the integral membrane bound anti-sigma factor RseA captures RpoE, retaining it to the inner membrane (De Las Peñas et al., 1997; Missiakas et al., 1997; Campbell et al., 2003). The stepwise cleavage of RseA is characteristic of RIP (Ehrmann and Clausen, 2004). The site-1 protease DegS senses misfolded C-terminal portions of OMPs with its PDZ-domain (Walsh et al., 2003; Wilken et al., 2004). In *V. cholerae*, OmpU is the essential stress sensor for membrane damaging and misfolded periplasmic proteins, e.g., in presence of antimicrobial peptides to activate a RpoE-dependent resistance. The signal transduction is based on the exposure of OmpU C-terminal YDF motifs, which interact with the PDZ-domain of DegS and hence activate RpoE activity (Mathur et al., 2007). DegS is a member of the DegS/HtrA2-subfamily of oligomeric serine HtrA proteases that possesses an N-terminal transmembrane portion, a catalytic serine protease domain and a C-terminal PDZ-domain (Clausen et al., 2002). DegS is the only known protease targeting RseA in *E. coli*. Additionally, in *V. cholerae* DegS also finds the sulfide-thiol reduced ToxR as its substrate (Lembke et al., 2018). DegS is activated by the interaction between C-terminal OMP peptides and its PDZ-domain. Refolding of DegS is induced resulting in proteolytic activity and the cleavage of RseA at its periplasmic portion, which triggers a second cut by the site-2 protease RseP at the cytoplasmic portion of RseA. RseP is a zinc metalloprotease of the inner membrane, harboring highly conserved HEXXH and LDG motifs and a PDZ-domain (Rudner et al., 1999; Kanehara et al., 2001; Drew et al., 2002). Eventually, RpoE is liberated into the cytoplasm where it assembles into the RNA polymerase holoenzyme. The first identified RpoE-dependent promoter in *V. cholerae* is located upstream of the *rpoErseABC* operon and harbors high similarity with consensus sequences of RpoE-regulated promoters in *E. coli* (Kovacikova and Skorupski, 2002). Comparative microarray analyses of a *rseA* deletion strain and wild type (WT) indicate that *degP* is also under RpoE-control in *V. cholerae* (Ding et al., 2004). Additionally, in a *degS* deletion strain less RpoE is released from RseA and consequently the RpoE-response is decreased, resulting in significantly reduced transcription of *degP* compared to WT (Lembke et al., 2018).

DegP is a periplasmic heat-shock protein, which is highly conserved across species and can act as both chaperone and protease (Spiess et al., 1999). DegP belongs to the HtrA-family of PDZ-domain containing proteases (Kolmar et al., 1996; Krojer et al., 2008a). Its structure is formed by trimer subunits that assemble to proteolytically inactive hexamers (Krojer et al., 2002). In presence of unfolded protein substrates, active DegP builds up dodecamers or icosatetramers (Jiang et al., 2008; Krojer et al., 2008b).

The type II secretion system (T2SS) of *V. cholerae*, encoded by two different *eps* operons (Sandkvist, 2001), is required for the secretion of enzymes and cholera toxin into the environment (Korotkov et al., 2012). Interestingly, a deletion of *eps* genes causes outer membrane damage which in turn activates RpoE-dependent response (Sikora et al., 2007). Furthermore, RpoE is also responsible for expression of the T2SS in *V. cholerae*, essential for release of important effectors along the lifecycle like the CTX or biofilm adhesion factors (Zielke et al., 2014). Concordantly, *rpoE* deletion strains are significantly attenuated in the murine model compared to WT (Kovacikova and Skorupski, 2002).

## The Alternative Sigma Factor RpoS (σ^S^ or σ^38^) Is Targeted by the ClpXP Protease

RpoS is a hallmark of a proteolysis-controlled regulator and was mainly characterized in *E. coli* to be responsible to counteract starvation conditions (Hengge-Aronis, 2002). Degradation of RpoS is under control of its specific proteolysis targeting factor, termed RssB (Muffler et al., 1996), which is activated by the kinase ArcB (Mika and Hengge, 2005) leading to a proteolytic complex comprising ClpXP, phosphorylated RssB, and RpoS (Becker et al., 1999; Zhou et al., 2001; Stüdemann et al., 2003). Anti-adaptor proteins, termed Ira (inhibitor of RssB activity) are identified to block RssB activity, thus stabilizing RpoS. Ira proteins (Battesti et al., 2013) respond to specific physiological stress conditions (Hryckowian et al., 2014), as well as to the accumulation of intracellular metabolites (Battesti et al., 2015). In *V. cholerae* the role of RpoS is less clear as a *rpoS-*mutant only exhibits minor defects in intestinal colonization (Yildiz and Schoolnik, 1998; Merrell et al., 2000). However, the “mucosal escape” of *V. cholerae* at late stages of the infection depends on RpoS regulated gene expression (Nielsen et al., 2006). This phenotype is marked by activation of chemotaxis and motility genes resulting in detachment from the mucosal surface and entrance into the gastrointestinal lumen (Nielsen et al., 2006). While flagellar motility is crucial to direct *V. cholerae* to the mucosal layer, the bacteria enter a non-motile state upon mucosal penetration (Liu et al., 2008). After infection progression, nutrients in the gut decline, thereby starvation and high cell density may trigger *rpoS* expression. RpoS activates chemotaxis and motility gene expression, subsequently resulting in the mucosal escape phenotype. Moreover, RpoS is responsible for biofilm dispersal in a hydrodynamic model (Müller et al., 2007) or for biofilm escape (Wurm et al., 2017).

Termination of the RpoS-program is less clear, but likely involves proteolytic control (Wurm et al., 2017). Under laboratory conditions, RpoS-levels can increase if bacteria are stressed by a shift from rich into poor nutrient conditions. The enhanced ppGpp concentrations activate *rpoS* transcription leading to high RpoS-levels, which in turn activate chemotaxis and motility gene expression (Wurm et al., 2017). Interestingly, as soon as ppGpp-levels decline, *rpoS* transcription stops and RpoS-proteolysis is subsequently activated by a RssB homolog and the ClpXP-protease complex (Wurm et al., 2017).

To date, *rpoS* expression control seems to be conserved in *Enterobacteriacae* and other Gram-negative bacteria (e.g., *Vibrio* and *Pseudomonas*). Stringent control, mRNA stability and ClpXP- or Lon-mediated proteolysis control *rpoS* expression levels. In contrast, RpoS-mediated responses are completely different in such species, leading to physiological changes like persistence, motility, and virulence (Schellhorn, 2014).

## Concluding Remarks

While transcriptional regulation along the lifecycle of *V. cholerae* has been intensively studied, we are just beginning to identify and understand post-translational control elements. Recent reports highlight that regulated proteolysis of alternative sigma factors or transcriptional regulators is involved in blockage or termination of gene expression comprising virulence, transmission, and environmental persistence conditions.

Regulated proteolysis events during *V. cholerae*'s lifecycle are summarized in Table 1. To conclude, FliA proteolysis ensures full virulence induction during initial colonization upon penetration of *V. cholerae* through the intestinal mucus. Meanwhile, the virulence cascade starts to operate, i.e., the ToxT positive forward loop regulation is activated. At some point during the end of the colonization phase and shortly before onset of mucosal escape, this virulence regulatory loop needs to be terminated via proteolysis of TcpP, ToxR, and ToxT (Figure 1 and Table 1). During colonization stage, RpoE is released via RIP of RseA and mediates host protective strategies. Regulated proteolysis maintains low RpoS levels during colonization, while starvation conditions inhibit RpoS proteolysis via RssB. Elevated RpoS levels at late infection stages subsequently initiate the mucosal escape mechanism.

Several important questions remain to be addressed to understand such complex post-translational regulation events. For example, what are the specific triggers initiating or preventing regulated proteolysis? What are the associated anti-proteolytic or targeting proteolysis factors, which protect proteins from degradation or attract key-proteases such as Lon, ClpXP, and DegS? With FlgM, RssB, RseA, ToxS, or TcpH probably only a fraction of such factors have been identified. Due to established intestinal infection and environmental persistence assays as well as its rapid proliferation requiring fast adaptation, *V. cholerae* is a valuable model to study and identify the proteolytic regulatory networks to gain deeper insights into the pathogen's bacterial physiology.

## Author Contributions

NP wrote parts of the RpoE chapter and RpoS chapter as well as designed Figure 1. ML wrote parts of the ToxR chapter. KP wrote parts of the introduction and FliA chapter. JM wrote the Tcp chapter. JR wrote parts of the RpoE, RpoS, ToxR chapter, and the ToxT chapter. SS was the coordinating author involved in writing and editing of all chapters as well as finalizing the manuscript for submission.

### Conflict of Interest Statement

The authors declare that the research was conducted in the absence of any commercial or financial relationships that could be construed as a potential conflict of interest.
